# Editorial: Gut Microbiome Modulation in Ruminants: Enhancing Advantages and Minimizing Drawbacks

**DOI:** 10.3389/fmicb.2020.622002

**Published:** 2021-01-11

**Authors:** Alejandro Belanche, Amlan K. Patra, Diego P. Morgavi, Garret Suen, Charles J. Newbold, David R. Yáñez-Ruiz

**Affiliations:** ^1^Estación Experimental del Zaidín, Consejo Superior de Investigaciones Científicas, Granada, Spain; ^2^Department of Animal Nutrition, West Bengal University of Animal and Fishery Sciences, Kolkata, India; ^3^Université Clermont Auvergne, INRAE, VetAgro Sup, UMR 1213 Herbivores Unit, Saint-Genès-Champanelle, France; ^4^Department of Bacteriology, University of Wisconsin–Madison, Madison, WI, United States; ^5^Scotland's Rural College, Edinburg Campus, Edinburgh, United Kingdom

**Keywords:** diet, early life, feed additive, feed efficiency (FE), health, methane, rumen protozoa, rumen microbiome

Historically, the gastrointestinal tract (GIT) was considered an organ solely equipped for the digestion and absorption of nutrients. However, the GIT harbors the largest population of immune cells and microbes that outnumber the entire host cells. Therefore, there is a general consensus that a healthy gut leads to healthy ruminants with optimal performance. In this context, the rumen is perhaps the most diverse and complex microbial ecosystem harbored in the GIT of animals. This microbial community consisting of symbiotic bacteria, archaea, protozoa, fungi, and phages provides an evolutionary advantage for ruminants, which allows them to utilize lignocellulosic materials and non-protein nitrogen to produce high quality foods. As a result, ruminants are capable of digesting a wide range of forages, decreasing the competition for human-edible foods. However, rumen microbial fermentation has some drawbacks: proteolysis carried out by protozoa and certain bacterial species can lead to low nitrogen efficiency (Belanche et al., 2012) and the excess ruminal ammonia, not captured by the ruminal microbiota for their own protein synthesis, is absorbed and excreted to the environment. Similarly, CH_4_ formation in the rumen by the methanogenic archaea is wasteful in terms of feed energy loss as well as contributes to climate change. Moreover, rumen fatty acid biohydrogenation by rumen microbes leads to more saturated fat in ruminants' milk and meat in comparison to monogastric animals. Thus, understanding the interactions between the gut microbiome, diet, host genetics, and health are key to develop new strategies to meet consumers' demands for better food quality, animal health, and a more environmentally friendly and efficient animal production. This Research Topic aimed to propose nutritional and other rumen manipulation strategies and other insights to enhance the advantages, and to minimize the drawbacks, of the ruminant digestive physiology by modifications of the gut microbiome and its functionality.

The Research Topic compiled 29 original publications including 27 Original Research papers, one Mini Review, and one Hypothesis Theory article, as well as one Corrigendum. Most of these studies were conducted in China (*n* = 14), followed by Brazil (*n* = 3), United Kingdom (*n* = 3), Spain (*n* = 2), Germany (*n* = 2), and USA (*n* = 2), whereas Austria, Chile, and Ireland had one study each. Most of these publications consisted in *in vivo* studies (*n* = 20), while other used *in vitro* (*n* = 4), *in silico* (*n* = 1), meta-analysis (*n* = 1), and literature reviews and theoretical hypothesis-developing studies (*n* = 3) to describe the mode of action of various nutritional interventions. Some of these studies proposed novel strategies which lead to future research hypotheses. The publications benefited from using multidisciplinary approaches combining omics-based techniques to characterize the GIT microbiota to develop new nutritional interventions for desirable microbiome structure and function and to optimize productivity or to minimize the environmental impact of ruminant agriculture. The publications were classified in eight thematic areas: early life nutritional interventions (*n* = 4), gut microbiota and health (*n* = 3), diet composition (*n* = 5), feed additives (*n* = 4), feed efficiency (*n* = 4), rumen methanogenesis (*n* = 6), rumen protozoa (*n* = 2), and methods (*n* = 1), which are discussed below.

## Early Life Nutritional Interventions

The rumen microbiota in adult animals has been demonstrated to be highly redundant, resilient and host-specific (Weimer, 2015). As a result, once any nutritional intervention ceases, the rumen microbiota and its function return to the original state making it difficult to permanently modify a fully mature rumen microbiome in adult animals. The developing rumen of the newborn animals may represent an opportunity for microbial programming by modifying the type of microbial groups that first occupy the ecological niches in the rumen of young ruminants (Yáñez-Ruiz et al., 2015; Furman et al., 2020). This Research Topic contains some of the latest studies to understand the gut microbial colonization and ways to modify this process.

Li B. et al., provided a detailed description of the bacterial colonization process of the different segments of the GIT in young goats including the rumen, duodenum, jejunum, ileum, caecum, and colon. This study showed that the greatest increase in microbial diversity occurred between 14 and 28 days of age. Moreover, rumen microbiota was shown to be more sensitive to the introduction of solid feeds than the intestinal microbiota, opening the possibility to further explore ways for favorable rumen microbial manipulation during the weaning transition.

In this line, Cui, Wu, Li et al., showed that artificial rearing of young yak calves fed with milk replacer, starter feed, and alfalfa hay, in comparison to maternal rearing of yaks fed pasture, during the pre-weaning period is beneficial to the GIT development and its digestion, absorption, immune function, and animal performance. This observation, disagrees with previous findings that suggested that maternal rearing, in comparison to artificial milk feeding, accelerates the rumen microbiological and physiological development in lambs (Belanche et al., 2019b) with positive effects on animal performance (Belanche et al., 2019c). These results suggest that a limited availability and diversity of feed resources during the pre-weaning period (as usually occurs in young grazing yaks) can limit the GIT microbial development due to the insufficient nutrient supply for the rumen microbes. In a follow up study, Cui, Wu, Liu et al., built upon this hypothesis and showed that milk replacer supplemented with alfalfa hay and starter feeding during the pre-weaning period (in comparison to supplemented with alfalfa alone, starter alone, or un-supplemented) is beneficial to the GIT in terms of rumen microbiological and functional development (i.e., higher bacterial diversity, VFA concentration, and pectinase activity) and intestinal activity and immune function (i.e., higher α-amylase, trypsin, IL-1β, TNF-α, and IFN-β) in yak calves. The availability of different carbon and nitrogen sources from both fibrous and non-fibrous carbohydrates seemed to benefit the GIT microbial colonization as well as the rumen function (papillae development and fermentation) and intestine anatomical development (villus and crypt) leading to an enhanced animal growth.

Bu et al., investigated the possibility of improving animal performance and health through reprograming the rumen prokaryotic microbial assemblage of young calves by oral inoculation with rumen microbiota from adult cows. This inoculation decreased the incidence of diarrhea and the stochasticity of the rumen microbial community development, but it had no effects on the overall rumen microbiota, its functional profiles and the productivity outcomes. These observations disagree with previous positive effects of inoculation with adult rumen microbiota reported in lambs (Zhong et al., 2014; De Barbieri et al., 2015), goat kids (Belanche et al., 2020), and calves (Muscato et al., 2002) on the GIT microbial development and productivity. This discrepancy suggests that further research should focus on the type of source of microbial inocula (i.e., diet consumed by the donor animal), its preservation method (e.g., fresh vs. defrosted vs. lyophilized), inoculation frequency and time window to maximize the positive effects of such intervention (Belanche et al., 2019a).

## Gut Microbiota and Health

The GIT microbiome shields the host animal against environmental threats and diseases through various mechanisms including the modulation of the immune system and promoting health and productivity in ruminants (Celi et al., 2017). The perturbation of this commensal microbiota can result in GIT disorders such as rumen acidosis, bloat, nutrient toxicity, and diarrhea. These health problems, which represent major welfare and economic concerns in the current intensive production systems, have been conventionally addressed through the “one-pathogen one-disease” approach. However, there is increasing evidence showing the importance of symbiotic microbiomes as major players modulating and minimizing the incidence of these GIT disorders as well as mastitis and respiratory disease (Mao et al., 2015). Although in its infancy, microbial endocrinology studies the ability of microorganisms to produce and respond to neurochemicals that originate either within the microbes or within the host they inhabit. This crosstalk between the microbes and their host gives new insights into the ways the GIT microbiota can affect host stress, metabolic efficiency, or resistance to disease. This approach could potentially translate into new management practices such as feeding diets with low levels of neurochemical precursors (e.g., tyrosine) to minimize stress and aggressiveness (Lyte et al., 2018).

Within this Research Topic, Wang Y. et al., compared the microbial populations from feces in healthy and diarrheic kids as well as in healthy adult goats. Using 16S rRNA sequencing, this study showed that healthy kids and adult goats share a similar rumen bacterial composition, whereas diarrheic kids had a significantly different rumen microbiota. This observation suggests that the higher susceptibility of the kids to diarrhea, in comparison to adult animals, is not associated with the initial composition of their GIT microbiota and is more likely to be caused by other factors. However, when diarrhea is established, for whatever reason, it exacerbates the intestinal microbiota promoting a decrease in the species richness and diversity. This study also identified several bacterial species which can be considered as indicators of a healthy intestinal microbiota providing theoretical basis for the prevention and treatment diarrhea.

The study of Zhong et al., is the first of its kind to investigated the link between rumen microbiota and the milk Somatic Cells Counts (SCC) as an indicator of mastitis and inflammatory disease in cows. After studying the rumen microbiome in SCC divergent cows, it was observed that the cows with high SCC (3,107,610 cells/ml), in comparison to those with low SSC (71,460 cells/ml), showed poorer milk yield, milk composition, and lower rumen VFA concentration, but also higher rumen bacterial diversity and increased numbers of phyla SR1, *Actinobacteria, Clostridiales*, and *Butyrivibrio*. This observation suggest that rumen microbial composition is linked, to some extent, to udders health as well as it has been correlated to milk yield (Shabat et al., 2016) and feed efficiency as described below. However, special caution should be taken about inferring causality since deficient management or hygiene practices (which led to health issues) and a poor nutrition (linked with rumen microbiota) are often associated in on farm conditions.

The effect of aging on the relation between gut microbiota and health has been studied in humans but little attention has been paid to it in livestock animals. The study of Zhang et al., is one of the first to explore the impact of aging in a large number of dairy cows across various farms. It was reported that old cows (>5 lactations), in comparison to medium-aged (3 lactations) and young cows (primiparous), suffered from long-term and low-level chronic inflammation as indicated by increased levels of inflammatory cytokines IL-10, TNF-α, TGF-β, and SCC, as well as a lower milk yield. This aging process decreased the abundance of beneficial bacteria such as Bacteroidaceae, Eubacterium, and *Bifidobacterium* in feces, as well as the metabolic functions related to carbohydrate and lipid metabolism. These observations open the possibility of programming the cows' longevity by manipulating the gut microbiota in early life. However, to successfully achieve this goal, a more holistic on farm approach should be considered including improvements in housing, nutrition, milking practices, herd management, genetic selection for longevity, and health programs (Essl, 1998) to discard potential associative effects as described before.

## Diet Composition

Diet composition and feed intake are some of the main drivers which determine not only the GIT microbiota but also productive outcomes in ruminants. Wang L. et al., after conducting a metagenomic analysis of the rumen microbiota in cows showed that high-forage diets, in comparison to those fed high-concentrate diets, promoted higher bacterial diversity and abundance of *Bacteroides, Fibrobacter*, and *Ruminococcus*. These microbes were also identified as the key contributors to the carbohydrate-active enzymes (CAZymes), such as cellulase enzymes, which allowed a more efficient degradation of plant cell wall polysaccharides in the rumen. Using a similar metagenomics analysis, Shen et al., reported that dairy goats fed high levels of rumen degradable starch, in comparison to low, had higher ruminal abundance of genes encoding for microbial amylases leading to higher propionate concentration. Feeding degradable starch also promoted lower levels of genes encoding for enzymes involved in cellulose degradation and starch branching which were mostly present in Prevotellaceae, Ruminococcaceae, and Bacteroidaceae. These two experiments highlighted the great differences in the rumen microbiota and its function according to the availability of fermentable carbohydrates.

In this sense, the sudden increase in the availability of highly fermentable carbohydrates when ruminants enter into a feedlot system represents a challenge for their GIT microbiota, which needs to be adjusted. Pinto et al., showed that Nellore bulls previously exposed to concentrate for at least 2 weeks, in comparison to those fed hay alone or restricted amounts of hay, seem to adapt better to high concentrate finishing diets leading to higher DM intake, propionate molar proportion and lower bacterial diversity, phenomena that have previously been associated with higher feed efficiency (Shabat et al., 2016). The opposite dietary shift from a concentrate-rich to a grazing diet was studied by Belanche et al., in sheep including a detailed multi-kingdom characterization of the rumen microbiota. This microbial adaptation to grazing conditions required an increase in the microbial concentration (bacteria, methanogens, and protozoa), diversity (bacteria, methanogens, and fungi), microbial network complexity, and abundance of key microbes involved in cellulolysis, proteolysis, lactate production, and methylotrophic archaea. This multi-factorial microbial adaptation indicated that pasture degradation is a complex process which requires a diverse consortium of microbes working together.

Beyond the type of carbohydrates, Li Z. et al., revealed that the level of nitrogen supplementation also has a modulatory effect on the rumen bacteria present in the solid, liquid, and epithelium-associated fractions. Authors noted that urea supplementation increased the ruminal concentrations of ammonia and butyrate in detriment of propionate concentration. Moreover, the greatest differences were observed among the three bacterial fractions (liquid, solid, and epithelium-associated microbiota), with the epithelium-associated microbial communities being the most reactive to urea supplementation due to their taxonomic and functional peculiarities for regulating urea recycling via ruminal epithelium.

## Feed Additives

Novel and cost-effective strategies must be explored to modulate the rumen microbial fermentation since antibiotics are being phased out as growth promoters in livestock production. In recent years, the exploration of bioactive phytochemicals as natural feed additives and probiotics has gained interest to favorably modify the rumen fermentation (Patra and Saxena, 2009). Nevertheless, the effectiveness of these feed additives is highly variable depending upon the nature of the compound, its concentration in the plant, the basal diet consumed by the animal and the potential adaptability of the rumen microbiota.

Petri et al., explored the dietary supplementation with autolyzed yeast (*Saccharomyces cerevisiae*) or with phytogenics (a blend of spices, herbs, and essential oils) as a way to prevent subacute rumen acidosis in dairy cows. These authors noted that the addition of phytochemicals tended to impact the rumen epithelial microbiota, whereas autolyzed yeast tended to impact gene expression in the rumen epithelium. This study indicated that a better understanding of the relationships between the rumen epithelial microbiota, the diet and the production of biogenic amines and endotoxins is necessary to develop future strategies to prevent rumen acidosis.

Hu et al., conducted a sophisticated experiment in which yaks under a growth-retardation situation were fed the same basal diet alone or supplemented with either cysteamine hydrochloride or active dry yeast. Both feed additives showed positive effects on the rumen function such as upregulation of genes related with VFA absorption and compensatory growth as a result of greater nutrient intake or feed efficiency. Authors showed that the abundance of certain beneficial bacteria such as *Fibrobacter, Treponema, Butyrivibrio*, or *Prevotella* was positively associated with the tight junction function suggesting that regulating these bacteria may improve ruminal epithelial barriers in cattle fed concentrate rich diets. This holistic methodological approach highlighted the importance of the epithelial barrier for ruminal and overall health and productivity, which is detrimentally affected by various factors including acidosis and feed restriction in cows (Aschenbach et al., 2019).

Wang B. et al., also proposed a refined experimental setup including the use of amplicon sequencing and ultrahigh-performance liquid chromatography coupled to quadrupole time-of-flight mass spectrometry to describe the effect of diet supplementation with tea saponins on the rumen microbiome and metabolome in young cattle. These authors showed that tea saponins have the ability to modulate the ruminal microbiota and metabolites, despite having little impact on rumen fermentation. Interestingly, this study showed that the response was diet-dependent, having greater effects with soybean hulls compared to alfalfa hay diets, indicating that tea saponins cannot be consider a “one-fits-all” solution across diets. Similarly, Patra et al., explored the diet supplementation with menthol-rich plant bioactive lipids in a dose-response experiment to improve the rumen function in growing lambs. This nutritional intervention led to an increase in the diversity in the solid-associated bacterial community, a reduction of certain methanogen species and a small shift in the metabolic correlation networks, but it had no effect on the rumen fermentation pattern. As a result, authors hypothesized that stable rumen fermentation is maintained during minor microbial alterations as a result of the metabolic redundancy of the rumen ecosystem.

## Feed Efficiency

The rumen microbiome is tightly linked to the ruminant's ability to extract energy from feeds, termed feed efficiency. Previous publications (Shabat et al., 2016) suggested that lower rumen bacterial richness and CH_4_ emissions, along with higher propionate molar proportion and specific enrichment of microbes (e.g., *Megasphaera* and Lachnosiraceae) and metabolic routes (i.e., acrylate pathway) are distinctive aspects of feed efficient dairy cows. In the present Research Topic, Lopes et al., investigated the bacterial and fungal microbiota of the rumen (liquid and solid fractions), small intestine, caecum and feces in Nellore steers with divergent feed efficiency. This study concluded that fecal sampling can represent a non-invasive strategy to link the bovine microbiota with productivity phenotypes such as feed efficiency.

Using a metagenomics approach Auffret et al., provided more depth in the description of the ruminal mechanisms which determine the feed efficiency in beef cattle fed high concentrate diets. This study revealed that microbial species (e.g., *Eubacterium*) carrying genes involved in the feed adhesion, biofilm formation, and butyrate and propionate production in the rumen seem to be important mechanisms explaining significant differences in feed efficiency among animals. On the contrary, microbial mechanisms associated with low efficient animals involved the presence of potential pathogens (e.g., Proteobacteria and Spirochaetales) or production of acetate and inhibitors of the host immune system (e.g., sialic acid). These findings provide some insights to better understand the crosstalk between the microbiota and the host-animal through the rumen epithelium in order to improve feed efficiency.

Ahmad et al., used bacterial sequencing coupled with a transcriptomics analysis of the rumen epithelium to investigate the effect of the diet on the host-microbiota crosstalk and feed efficiency in yaks. Authors indicated that the increase in dietary energy level promotes a concomitant increase in the abundance of Firmicutes and Bacteroidetes, the accumulation of fermentation products and the overexpression of VFA transporter genes in the rumen epithelium leading to higher feed efficiency than those animals fed lower energy levels. McDermott et al., provided more arguments to describe the multi-factorial nature of the feed efficiency concept by using an *in vitro* model. After mixing rumen fluids divergent in terms of *in vitro* DM digestibility, authors concluded that removal of host control alone in the *in vitro* model is not sufficient to allow successful and consistent changes in the rumen microbiota and its activity after cross inoculation. So, it seems that along with host factors, there are individual factors within each community that prevent other microbes from establishing given the high resilience of the rumen microbial ecosystem (Weimer, 2015).

## Rumen Methanogenesis

Enteric CH_4_ production represents a social and environmental concern as well as an energy loss for the ruminant; therefore the development of CH_4_ mitigation strategies has become a research priority in ruminant nutrition (Patra et al., 2017). Using an *in vitro* batch culture model, Wang K. et al., investigated the shifts of hydrogen metabolism during the rumen methanogenesis in response to changes in the diet. These authors noted that the replacement of forage fiber with non-forage fiber in the diet promoted a substitution of Firmicutes by Bacteroidetes and of *Methanobrevibacter* by *Methanomassiliicoccus* resulting in a shift in the hydrogen flow toward propionate production which could favor productive outcomes. Using the rumen simulation semi-continuous culture technique, Eger et al., explored the use of Mootral^TM^, consisting of a combination of organosulphur compounds from garlic and flavonoids from bitter orange. This strategy effectively reduced CH_4_ production through the inhibition of *Methanobrevibacter* spp. and *Methanobrevibacter thaueri* SGMT clade, without impairing rumen fermentation or the bacterial community. Whereas, Granja-Salcedo et al., studied the long-term effects (13 months) of diet supplementation with encapsulated nitrate in grazing beef cattle. This latter study demonstrated that encapsulated nitrate decreased the abundance of *Methanobrevibacter* in the rumen, promoted fumarate-reducers and lactate-producers and decreased acetate molar proportion. This decrease in the acetate percentage may rely on an indirect effect derived from the nitrate toxicity on methanogens which shifted hydrogen flow toward more reduced VFA such as propionate and butyrate. Most interestingly, nitrate supplementation persistently decreased CH_4_ emissions (−39%) and increased animal growth (+6%) suggesting lower energy loss from rumen methanogenesis.

Lactic acid bacteria (LAB) have been successfully used to decrease diarrhea and to improve ruminal development and feed efficiency in young ruminants (Signorini et al., 2012). The use of LAB (predominantly *Lactobacillus*) in adult animals also seems to have some positive effects on animal health, performance and decreasing rumen methanogenesis (Jeyanathan et al., 2014), but results are highly variable. Doyle et al., conducted a Critical Review on the use of LAB to reduce CH_4_ production in ruminants. Based on the available studies, most of them *in vitro*, it was concluded that LAB can reduce CH_4_ production, with this effect being strain-dependent. However, it could not be elucidated whether LAB or their metabolites affect the methanogens themselves, or indirectly through changes in the other rumen microbes that produce the substrates necessary for methanogenesis. As a result, authors could not provide a conclusive recommendation on the use of LAB for adult ruminants in on-farm conditions.

Ungerfeld launched a Hypothesis Theory integrating the most recent discoveries to better understand the metabolic hydrogen flows that occur during rumen fermentation. This author suggested that the combination of inhibitors of methanogenesis with adequate additives along with substrates which act as hydrogen acceptors can be an effective approach to decrease CH_4_ production and simultaneously redirect metabolic hydrogen toward fermentation end products with a nutritional value for the host animal.

Martínez-Álvaro et al., used respiration chambers to measure CH_4_ emissions in 72 steers and investigated the rumen microbiological differences among the extreme animals, which were classified as high- or low-CH_4_ emitters (based on kg CH_4_/kg DMI). These authors proposed a novel approach based on metagenomics and co-occurrence network analyses of the rumen microbes and their genes to predict the functional niches. Authors noted that differences in rumen CH_4_ emissions are mostly driven by non-methanogenic microbial communities (e.g., diversity and abundances of *Fibrobacter, Butyrivibrio, Pseudobutyrivibrio*), their activities (e.g., carbohydrate and amino-acids degradation), and fermentation products (e.g., butyrate and formate), rather than being solely methanogens-driven. These findings confirm previous hypothesis on the multi-factorial nature of rumen methanogenesis and represent a step change in our understanding for future genetic selection programs.

## Rumen Protozoa

Rumen protozoa are inhabitants but not essential denizens in the rumen ecosystem as their elimination from the rumen has been proposed as a strategy to increase the nutrient flow to the small intestine and to decrease rumen ammonia production and methanogenesis (Newbold et al., 2015). In this sense, Dai and Faciola conducted a meta-analysis including 66 publications about the different strategies to reduce rumen protozoa. These authors indicated that supplemental phytochemicals such as saponins, tannins, and medium chain fatty acids can be effective in reducing CH_4_ production but only if the rumen protozoa numbers reached values below 7 Log_10_ cells/ml. However, some of these nutritional strategies had relevant drawbacks such as reducing feed digestibility (e.g., with tannins) or the rumen microbial adaptation to the additive (e.g., saponins) which can limit the persistency of the effects over long periods. As a result, future studies should focus on the identification of the active molecules, doses and dietary interactions with these feed additives to increase their effectiveness.

Park et al., proposed a novel strategy to inhibit rumen protozoal growth and activity based on the specific inhibition of their lysozyme and peptidases. Authors noted that a lysozyme inhibitor reduced rumen protozoal numbers and feed digestibility *in vitro*. On the contrary, peptidase inhibitors shifted the protozoal community and fermentation profiles without adverse effects on feed digestion or fermentation. Furthermore, they found that cysteine peptidase inhibitors were more effective than serine peptidase inhibitors at lowering ammonia production by rumen protozoa. These findings open new possibilities to improve nitrogen utilization by ruminants.

## Technological Advances to Understand the Rumen Microbiome

The onset of next generation sequencing has represents an opportunity to better describe the rumen microbiome and to infer its function. Although these studies are of great value, interpretation of the results generated across studies remains a challenge due to differences with respect to DNA extraction, primers, downstream computational analyses, and databases used as reference. López-García et al., conducted one of the few studies which compared the performance and robustness of two of the most common software (QIIME vs. Mothur) and databases (GreenGenes vs. SILVA) used in meta-taxonomic studies of the rumen microbiome. This study provides practical recommendations to future users and clearly indicated that further advances in computational tools and culturomics are needed to improve the completeness of the rumen databases. These advances will allow to better assess microbial taxonomy and to infer their metabolic functions.

## Conclusions

This compendium of articles provides newer concepts and understanding of nutritional interventions such as early-life nutritional strategies, diet composition, host genetics, and natural feed additives to modulate ruminal metabolic functions and microbial community, particularly methanogens, protozoa and cellulolytic bacteria, which could improve feed efficiency as well as ruminal and overall host health. Improved understanding of the alterations the GIT microbiota during metabolic disorders (e.g., rumen acidosis, diarrhea, mastitis) and stressful conditions (e.g., weaning or lactation peak) are needed to manage the host-microbiome interactions. This compendium of articles also highlights that the rumen microbial ecosystem has a great plasticity to adapt to multiple nutritional interventions, however this microbial community composition changes in a greater order of magnitude than the digestion and rumen fermentation does, evidencing functional redundancy among microbial species. Overall, this collection of articles provides a profound overview on the state of the art of the nutritional and rumen manipulation strategies studied in recent years in order to improve productivity and health as well as to minimize the environmental impact of the ruminant production. We are delighted to present this Research Topic and hope that it will promote further research in this area of gut microbiome linked to production and health of ruminants.

## Author Contributions

AB conceptualized and coordinated the Research Topic and wrote the manuscript. All authors edited this Research Topic and reviewed and approved this manuscript.

## Conflict of Interest

The authors declare that the research was conducted in the absence of any commercial or financial relationships that could be construed as a potential conflict of interest.
